# Effects of Psilocin and Psilocybin on Human 5-HT_4_ Serotonin and H_2_ Histamine Receptors in Perfused Hearts of Transgenic Mice

**DOI:** 10.3390/ph18071009

**Published:** 2025-07-06

**Authors:** Pauline Braekow, Joachim Neumann, Uwe Kirchhefer, Ulrich Gergs

**Affiliations:** 1 Institute for Pharmacology and Toxicology, Medical Faculty, Martin Luther University Halle-Wittenberg, 06097 Halle, Germany; pauline.braekow@student.uni-halle.de (P.B.); joachim.neumann@medizin.uni-halle.de (J.N.); 2 Institute of Pharmacology and Toxicology, University of Münster, 48149 Münster, Germany; kirchhef@uni-muenster.de

**Keywords:** psilocybin, psilocin, ergometrine, ergotamine, LSD, Langendorff heart

## Abstract

**Background/Objectives**: Hallucinogenic substances such as psilocybin, psilocin, ergometrine, ergotamine, and lysergic acid diethylamide (LSD) have been demonstrated to enhance the force of contraction (FOC), in part due to the phosphorylation of phospholamban in human atrial preparations via 5-HT_4_ serotonin receptors and/or H_2_ histamine receptors. However, whether psilocybin or psilocin acts at isolated mammalian ventricular preparations and whether they increase protein phosphorylation in the mammalian ventricle remains to be elucidated. **Methods**: To this end, the FOC and phospholamban phosphorylation in isolated perfused hearts from transgenic mice with cardiomyocyte-specific overexpression of either human 5-HT_4_ receptors (5-HT_4_-TG) or human H_2_ receptors (H_2_-TG) and their wild-type littermates (WT) were examined. Furthermore, the ergot alkaloids ergometrine, ergotamine, and LSD were used as references. **Results**: Psilocybin and psilocin enhanced the FOC to 137% and to 152%, respectively, and elevated the phospholamban phosphorylation in isolated perfused hearts from 5-HT_4_-TG. In H_2_-TG hearts, psilocybin and psilocin increased the FOC to a much lesser extent but had no effect on the phospholamban phosphorylation. In contrast, LSD increased the FOC and phosphorylation state of phospholamban in isolated hearts of both 5-HT_4_-TG and H_2_-TG. On the other hand, ergometrine and ergotamine increased the FOC only in H_2_-TG. Ergometrine increased the phosphorylation state of phospholamban in perfused hearts from H_2_-TG, but not from 5-HT_4_-TG. Ergotamine failed to increase the phospholamban phosphorylation in both H_2_-TG and 5-HT_4_-TG. Psilocybin, psilocin, ergotamine, ergometrine, and LSD were unable to increase FOC and phospholamban phosphorylation in perfused hearts from WT. **Conclusions**: The increase in the phosphorylation state of phospholamban could provide a partial explanation for the positive inotropic effects and the relaxant effects of not only psilocybin and psilocin but also ergometrine and LSD in the isolated hearts of the animals used in this study.

## 1. Introduction

Serotonin (5-hydroxytryptamine, 5-HT) and histamine are well-studied neurotransmitters that also exert direct inotropic and chronotropic effects on the human cardiovascular system [1,2,3,4,5,6,7,8]. 5-HT augments the force of contraction (FOC), shortens the time of relaxation, and increases the rate of relaxation in isolated electrically stimulated atrial preparations via human 5-HT_4_ receptors (reviewed in [2,9]). In the hearts of humans, monkeys, and pigs, but not of other mammals, 5-HT raises FOC via 5-HT_4_ receptors. In the hearts of wild-type mice (WT), 5-HT does not affect the FOC due to the absence of functional 5-HT receptors. Therefore, a transgenic mouse model with cardiac myocyte-specific overexpression of the human 5-HT_4_ receptor (5-HT_4_-TG) has been developed [10]. Regarding this mouse model, 5-HT raised FOC in isolated perfused hearts from 5-HT_4_-TG, but not in cardiac preparations from WT littermate mice, as demonstrated, e.g., in [10]. Interestingly, a similar situation can be found for histamine. In the human heart, histamine is capable of increasing the FOC via the stimulation of H_2_ histamine receptors [1,11]. Again, in WT mouse hearts, a direct inotropic effect of histamine via histamine receptors is missing, and therefore, a transgenic mouse model with cardiac myocyte-specific overexpression of the human H_2_ histamine receptor (H_2_-TG) has been generated to obtain a suitable animal model for studying the cardiac role of H_2_ receptors [12].

Psilocin is used in some regions of the world in religious ceremonies and in some regions as a recreational substance [9]. More recently, clinical interest in psilocin has grown for the treatment of psychiatric diseases [13,14,15,16]. In general, major depressive disorders (especially therapy-resistant depression), mood disorders, anxiety disorders, post-traumatic stress disorders, substance-use disorders, and dementia are significant issues in our society, underscoring the need for new therapeutic approaches. The use of psychedelic substances for the treatment of therapy-resistant depression is therefore increasingly coming into focus [17,18]. The psychedelics under investigation, such as lysergic acid diethylamide (LSD), psilocybin, mescaline, or N,N-dimethyltryptamine, are primarily agonists at the neuronal serotonin 5-HT_2A_ receptor. The extent to which peripheral serotonin receptors or other receptors play a role has not yet been fully elucidated. One key point to consider is the potential side effects of psychedelics based on neuronal and peripheral receptor binding. In recent studies, mainly neuronal side effects have been reported [19]. The majority of reviews on the subject indicate a need for additional research on the tolerability and side effects of psychedelics (e.g., Refs. [17,18,19]). In the case of psilocybin, several controlled studies have demonstrated its efficacy in the psilocybin-assisted therapy of major depressive disorders and other psychiatric disorders [20,21]. Moreover, these studies have shown that psilocybin is well-tolerated and toxicologically safe [20,21]. Some cardiovascular side effects, such as tachycardia and increased blood pressure, were reported [22]. It is noteworthy that ECG recordings revealed QTc interval prolongation, particularly at high doses of psilocybin or psilocin [23]. However, this prolongation appeared not to be hERG channel-mediated [24]. This suggests that further investigation of the cardiac effects of psilocybin and psilocin is warranted. Consequently, this was the aim of the present study.

In a similar fashion, LSD has been tried in the past in psychiatry but was forgotten. Currently, LSD is undergoing a revival as a therapeutic drug in psychiatry [16,25,26]. The natural alkaloids ergotamine and ergometrine, which are derived from ergot fungi, have been used in medicine in the past to treat migraine or increase blood pressure, respectively, but have fallen out of interest because drugs with fewer side effects were developed [27,28]. In addition to psilocin, the present study also focused on psilocybin, as it is believed to be the prodrug of psilocin and is normally considered inactive [29,30]. Furthermore, ergometrine and ergotamine were also examined due to their structural similarity to LSD in order to gain insights into the structure–effect relationship of these compounds. As demonstrated recently, psilocin and psilocybin increased FOC and the phosphorylation of phospholamban in atrial preparations of 5-HT_4_-TG [31], but their action in the ventricle of 5-HT_4_-TG remains unreported.

LSD and ergotamine increased FOC in isolated atrial preparations from H_2_-TG and 5-HT_4_-TG but not WT, suggesting that these effects are mediated by two different receptors [32,33]. This was accompanied by an increase in phospholamban phosphorylation in left atrial preparations from H_2_-TG and 5-HT_4_-TG. In contrast, ergometrine increased the force, contraction, and phosphorylation state of phospholamban in atrial preparations from H_2_-TG and not from 5-HT_4_-TG [34]. However, the effect of LSD, ergotamine, and ergometrine on the phosphorylation state of phospholamban in the whole mouse heart from H_2_-TG and 5-HT_4_-TG has not yet been reported and has now been examined in this study.

The present study was designed to examine the contractile effects and the effect on the phosphorylation state of phospholamban of psilocybin and psilocin in the isolated perfused hearts of H_2_-TG and 5-HT_4_-TG compared to WT hearts. This study was also conducted to investigate a possible link between these receptors and the cardiovascular side effects of psilocybin or psilocin. For comparison, the effects of LSD, ergotamine, and ergometrine were also studied.

## 2. Results

### 2.1. Force of Contraction

**Figure 1:**
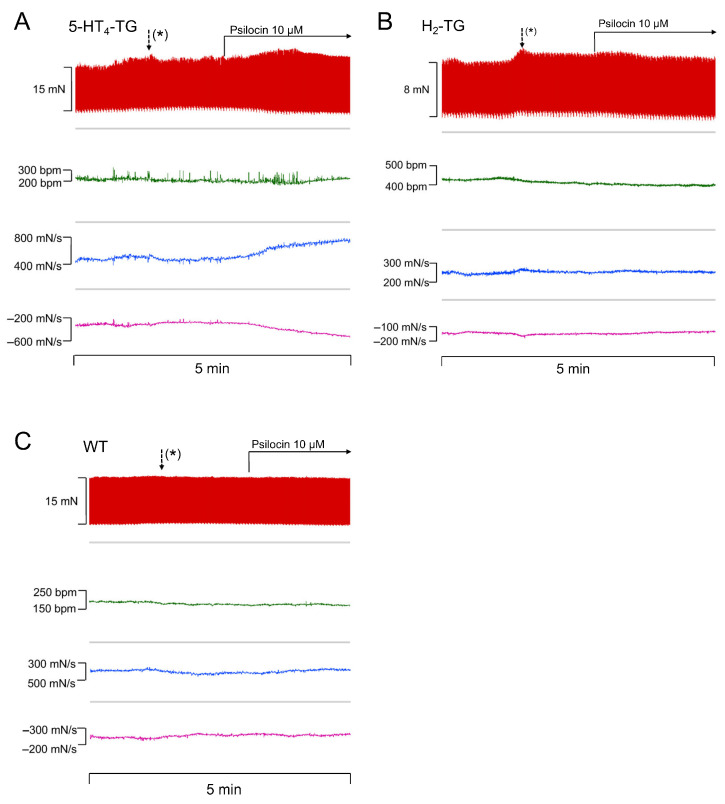
Psilocin increases force of contraction (FOC) in perfused mouse hearts. Original recordings of the effect of psilocin on FOC in milli Newton (mN), beating rate in beats per minute (bpm), maximum rate of tension development in milli Newton per second (mN/s), and minimum rate of tension relaxation in mN/s in 5-HT_4_-TG (**A**), H_2_-TG (**B**), and WT (**C**). (*) Activation of the syringe pump: after approximately one minute, the infused substances reach the heart.

Isolated spontaneously beating perfused mouse hearts from WT and transgenic mice were prepared according to the Langendorff method, and the effect on the FOC was measured for psilocin and psilocybin, as well as for ergot alkaloids (ergometrine, ergotamine, LSD). In original recordings, it is demonstrated that psilocin increased the rate of tension development and relaxation in 5-HT_4_-TG (Figure 1A) but not in H_2_-TG (Figure 1B) and WT (Figure 1C). Several such experiments for psilocin on contractile parameters in H_2_-TG, 5-HT_4_-TG, and WT are presented in Table 1. For instance, it was determined that psilocin increased the FOC in isolated Langendorff hearts of 5-HT_4_-TG from 12.1 ± 1.9 to 18.4 ± 1.5 mN (by about 60%) and the rate of tension development from 332.3 ± 41.1 to 536.8 ± 52.3 mN/s. The rate of relaxation increased from −230.1 ± 32.0 to −381.5 ± 34.6 mN/s. In addition, as FOC increased, the beating rate decreased from 332.9 ± 28.2 to 275.0 ± 35.6 bpm (Table 1, *p* < 0.05). Interestingly, psilocin administration resulted in a significant augmentation of approximately 13% of the FOC in Langendorff-perfused hearts from H_2_-TG (Table 1). However, psilocin failed to increase the rate of tension development, rate of relaxation, and beating rate in H_2_-TG and was inactive in Langendorff-perfused hearts from WT mice (Table 1).

The administration of psilocybin led to an increase in the FOC (by approximately 38%), the rate of force development, and the rate of relaxation in isolated Langendorff-perfused hearts from 5-HT_4_-TG. In contrast to psilocin, psilocybin failed to decrease the beating rate in whole hearts (Table 2). Like psilocin, psilocybin was also inactive in Langendorff-perfused hearts from WT mice (Table 2). Similar to psilocin, psilocybin has been observed to enhance the FOC in Langendorff-perfused hearts from H_2_-TG. Although the observed effect was only 11%, it was statistically significant (Table 2).

The next step was to analyze the ventricular effects of the ergot alkaloids. Interestingly, LSD increased the FOC and the rate of tension development in both H_2_-TG and 5-HT_4_-TG. In addition, LSD augmented the rate of relaxation and time to peak tension in H_2_-TG, but not 5-HT_4_-TG. Like psilocin, LSD decreased the beating rate while increasing the FOC (Table 3, *p* < 0.05). LSD was inactive in Langendorff-perfused hearts from WT mice (Table 3).

Ergometrine elevated the FOC, the rate of force development, and the rate of relaxation in isolated Langendorff-perfused hearts from H_2_-TG. Moreover, ergometrine reduced the time of relaxation in H_2_-TG, but did not alter the beating rate (Table 4, *p* < 0.05). However, ergometrine was inactive in Langendorff-perfused hearts from WT mice and 5-HT_4_-TG (Table 4).

Finally, ergotamine augmented the FOC, the rate of force development, and the rate of relaxation in isolated Langendorff-perfused hearts from H_2_-TG. Ergotamine failed to shorten the time to peak tension and time of relaxation and did not alter the beating rate (Table 5). Moreover, ergotamine was inactive in Langendorff-perfused hearts from WT mice and in 5-HT_4_-TG (Table 5).

### 2.2. Protein Phosphorylation

**Figure 2:**
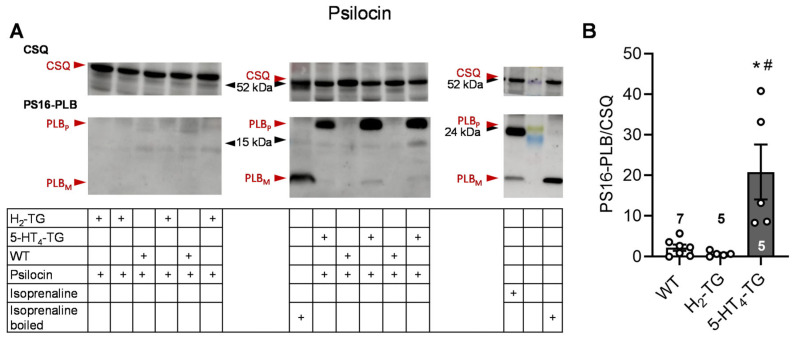
Effects of psilocin on phosphorylation in the perfused heart. Effects of 10 µM psilocin on phosphorylation of phospholamban (PLB) at amino serine 16 (PS16-PLB) in isolated spontaneously beating whole hearts from wild-type (WT), 5-HT_4_-TG, and H_2_-TG. Typical Western blots are seen in (**A**). Western blots depict high (p, pentameric) and low molecular (m, monomeric) weight forms of PLB labeled with arrows. The molecular weight reduction in boiled isoprenaline samples, which is characteristic of PLB, is shown on the right blot. As a loading control, the protein expression of calsequestrin (CSQ, indicated with an arrow) was utilized by cutting the lanes of the blot and incubating the lower and upper halves with different primary antibodies. (**B**) The ratios of the signals for PS16-PLB and the corresponding CSQ are shown. To compare WT, H_2_-TG, and 5-HT_4_-TG, a one-way analysis of variance (ANOVA) was performed with Bonferroni correction. The dots represent the individual values, and the bars represent the means ± standard error of the mean. * *p* < 0.05 vs. WT; # *p* < 0.05 vs. H_2_-TG; numbers in bars = number of experiments.

**Figure 3:**
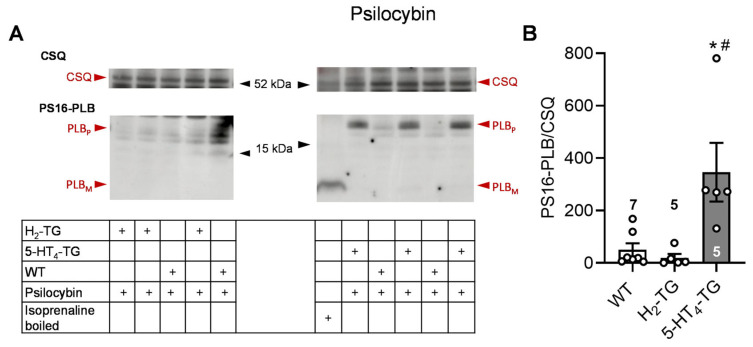
Effects of psilocybin on phosphorylation in the perfused heart. Effects of 10 µM psilocybin on phosphorylation of phospholamban (PLB) at amino serine 16 (PS16-PLB) in isolated spontaneously beating whole hearts from wild-type (WT), 5-HT_4_-TG, and H_2_-TG. Typical Western blots are seen in (**A**). Western blots depict high (p, pentameric) and low (m, monomeric) molecular weight forms of PLB labeled with arrows. As a loading control, the protein expression of calsequestrin (CSQ, indicated with an arrow) was utilized by cutting the lanes of the blot and incubating the lower and upper halves with different primary antibodies. (**B**) The ratios of the signals for PS16-PLB and the corresponding CSQ are shown. To compare WT, H_2_-TG, and 5-HT_4_-TG, a one-way analysis of variance (ANOVA) was performed with Bonferroni correction. The dots represent the individual values, and the bars represent the means ± standard error of the mean. * *p* < 0.05 vs. WT; # *p* < 0.05 vs. H_2_-TG; numbers in bars = number of experiments.

To better understand the underlying mechanisms of the contractile effects of psilocybin and psilocin, the protein phosphorylation of phospholamban on serine 16 was investigated by Western blotting. In previous studies, it was demonstrated that 5-HT or histamine could elevate the phosphorylation state of phospholamban in cardiac preparations from 5-HT_4_-TG or H_2_-TG, respectively, but not from WT [10,12]. In this context, the findings revealed that 1 µM of psilocin and psilocybin increased the phosphorylation state of phospholamban in isolated hearts from 5-HT_4_-TG but not H_2_-TG and WT. Original Western blots and summarizing scatterplots are depicted in Figure 2 for psilocin and in Figure 3 for psilocybin.

**Figure 4:**
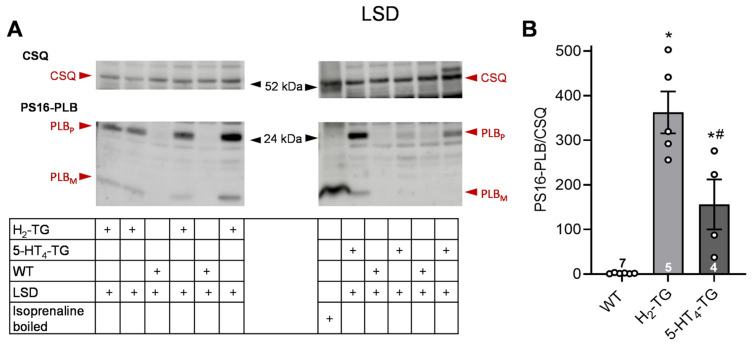
Effects of LSD on phosphorylation in the perfused heart. Effects of 10 µM LSD on phosphorylation of phospholamban (PLB) at amino serine 16 (PS16-PLB) in isolated spontaneously beating whole hearts from wild-type (WT), 5-HT_4_-TG, and H_2_-TG. Typical Western blots are seen in (**A**). Western blots depict high (p, pentameric) and low (m, monomeric) molecular weight forms of PLB labeled with arrows. As a loading control, the protein expression of calsequestrin (CSQ, indicated with an arrow) was utilized by cutting the lanes of the blot and incubating the lower and upper halves with different primary antibodies. (**B**) The ratios of the signals for PS16-PLB and the corresponding CSQ are shown. To compare WT, H_2_-TG, and 5-HT_4_-TG, a one-way analysis of variance (ANOVA) was performed with Bonferroni correction. The dots represent the individual values, and the bars represent the means ± standard error of the mean. * *p* < 0.05 vs. WT; # *p* < 0.05 vs. H_2_-TG; numbers in bars = number of experiments.

**Figure 5:**
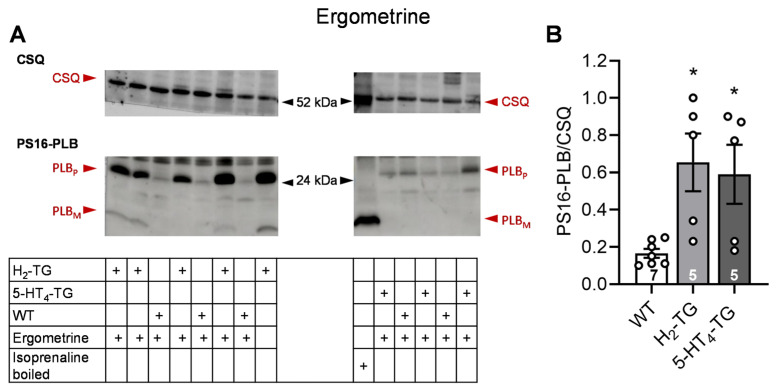
Effects of ergometrine on phosphorylation in the perfused heart. Effects of 10 µM ergometrine on phosphorylation of phospholamban (PLB) at amino serine 16 (PS16-PLB) in isolated spontaneously beating whole hearts from wild-type (WT), 5-HT_4_-TG, and H_2_-TG. Typical Western blots are seen in (**A**). Western blots depict high (p, pentameric) and low (m, monomeric) molecular weight forms of PLB labeled with arrows. As a loading control, the protein expression of calsequestrin (CSQ, indicated with an arrow) was utilized by cutting the lanes of the blot and incubating the lower and upper halves with different primary antibodies. (**B**) The ratios of the signals for PS16-PLB and the corresponding CSQ are shown. To compare WT, H_2_-TG, and 5-HT_4_-TG, a one-way analysis of variance (ANOVA) was performed with Bonferroni correction. The dots represent the individual values, and the bars represent the means ± standard error of the mean. * *p* < 0.05 vs. WT; numbers in bars = number of experiments.

**Figure 6:**
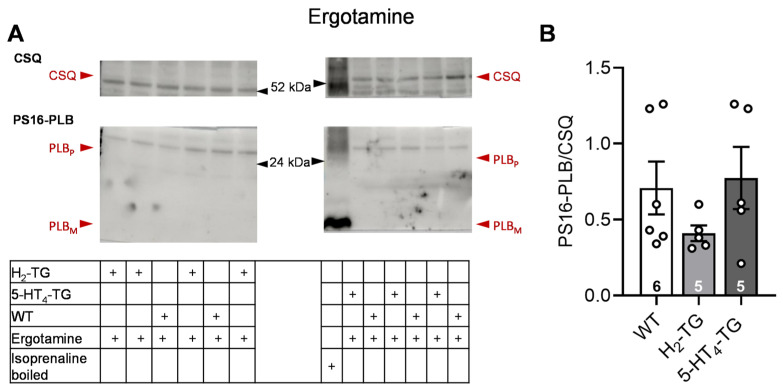
Effect of ergotamine on phosphorylation in the perfused heart. Effects of 10 µM ergotamine on phosphorylation of phospholamban (PLB) at amino serine 16 (PS16-PLB) in isolated spontaneously beating whole hearts from wild-type (WT), 5-HT_4_-TG, and H_2_-TG. Typical Western blots are seen in (**A**). Western blots depict high (p, pentameric) and low (m, monomeric) molecular weight forms of PLB labeled with arrows. As a loading control, the protein expression of calsequestrin (CSQ, indicated with an arrow) was utilized by cutting the lanes of the blot and incubating the lower and upper halves with different primary antibodies. (**B**) The ratios of the signals for PS16-PLB and the corresponding CSQ are shown. To compare WT, H_2_-TG, and 5-HT_4_-TG, a one-way analysis of variance (ANOVA) was performed with Bonferroni correction. The dots represent the individual values, and the bars represent the means ± standard error of the mean. Numbers in bars = number of experiments.

According to the contraction data, it was observed in H_2_-TG that LSD (Figure 4) and ergometrine (Figure 5) raised the phosphorylation state of phospholamban as seen in original Western blots and summarized in scatterplots. Additionally, LSD increased phospholamban phosphorylation in preparations from 5-HT_4_-TG. However, ergotamine failed under the same experimental conditions to raise the phospholamban phosphorylation (Figure 6).

## 3. Discussion

The main new finding in the present study is that psilocin and psilocybin increased both the force of contraction and phospholamban phosphorylation in the isolated hearts of 5-HT_4_-TG but not in hearts from H_2_-TG or WT. Previously, it has been reported that psilocin and psilocybin increased the FOC in left atrial preparations of these mice. However, the investigation did not include an assessment of ventricular function in WT and 5-HT_4_-TG mice [9].

Consequently, the present study has expanded the scope of prior investigations on psilocin from the mouse atrium to the mouse ventricle, a more physiological preparation than the isolated right atrium. In a previous study, psilocin was found to increase the beating rate in preparations of the right atrium [31]. In contrast, psilocin exhibited a negative chronotropic effect in the isolated whole heart, underscoring the putative importance of studying whole hearts that can behave differently from isolated auricles due to intact nervous pathways.

Likewise, psilocybin increases the beating rate in right atrial preparations from 5-HT_4_-TG [31] but not in isolated perfused hearts from 5-HT_4_-TG. In contrast, psilocin reduced the beating rate while increasing the FOC in 5-HT_4_-TG. The PIE of psilocin was previously only reported in the atrium of 5-HT_4_-TG [31]. However, for the output of blood from the heart, the ventricle is more relevant than the atrium. Therefore, one important finding of the present study is that it could be shown that ventricular contractility was also increased by psilocin, not only the atrial contractility as previously described [31].

In addition, psilocin has been demonstrated to increase the FOC in human atrial preparations [31]. However, due to the lack of access to human ventricular tissue, these human atrial studies could not be extended to the human ventricle. As a surrogate for the human ventricle, the FOC was measured in the mouse ventricle. These data could be used by others as a stimulus to study the contractile effects of psilocin in the human ventricle.

Another new and unexpected finding was that psilocin and psilocybin raised the FOC (by about 13% and 11%, respectively) also in the ventricle of H_2_-TG. This possibility has not yet been investigated in the atrium of H_2_-TG [31]. It can occur (e.g., noradrenaline on α-adrenoceptors and on β-adrenoceptors) that a substance acts on more than one receptor. Therefore, it would be quite possible that psilocin or psilocybin, like LSD [33], could have a PIE in the ventricle of both H_2_-TG and 5-HT_4_-TG, which seemed to be the case. Additionally, one interpretation of this finding is that the small PIE of psilocin and psilocybin in the H_2_-TG heart is quite specific, given that the PIE does not occur in WT. This might be of importance, as in the human heart, both receptors H_2_ and 5-HT_4_ are present. On the other hand, the potential involvement of hitherto unidentified processes could also play a role, as the phosphorylation of phospholamban was not affected by psilocin or psilocybin in H_2_-TG. Consequently, the results in H_2_-TG should be interpreted with caution.

Moreover, it was noted that ergometrine, like LSD, increased the phosphorylation state of phospholamban in the mouse ventricle of H_2_-TG. However, in contrast to LSD, ergometrine failed to increase FOC in the ventricle of 5-HT_4_-TG. Hence, a new finding here is that contractile specificity in FOC and biochemical increases in phosphorylation go hand in hand for ergometrine and LSD. Like above for psilocybin, a discrepancy between whole hearts and atrial preparations was apparent: ergometrine was as effective as histamine at 10 µM to increase the beating rate in atrial preparations from H_2_-TG [34]. In contrast, in whole hearts from H_2_-TG, ergometrine failed to exert a positive chronotropic effect. In a similar fashion, LSD increased the beating rate in isolated right atrial preparations from both 5-HT_4_-TG and H_2_-TG [33], but not in perfused hearts from 5-HT_4_-TG and H_2_-TG. If anything, the mean values for the beating rate were lower in the presence than in the absence of LSD in both genotypes.

The situation is more complicated in the case of ergotamine. In the 5-HT_4_-TG atrium, ergotamine increased the FOC only to a very small extent. In the WT atrium, ergotamine did not affect the FOC. It was therefore assumed that ergotamine acts as a partial agonist on 5-HT_4_ receptors [32]. In contrast, ergotamine seems to be a full agonist compared to histamine in the H_2_-TG atrium (Table 3, [32]). Moreover, the same tendency is observed in the ventricle: ergotamine is effective in raising the FOC in H_2_-TG but not in 5-HT_4_-TG. In addition, even in the ventricle from H_2_-TG where ergotamine raised the FOC, ergotamine failed to increase the phospholamban phosphorylation. It can be assumed that the efficacy of ergotamine is lower in the whole heart from 5-HT_4_-TG. This hypothesis is supported by the finding that ergotamine failed to shorten the time of relaxation, which is a very sensitive parameter that correlates with the phosphorylation of phospholamban at amino acid 16 [35]. In the isolated left atrium from 5-HT_4_-TG, it was observed that ergotamine only marginally increased the FOC. Furthermore, ergotamine increased the phosphorylation of phospholamban to a lesser extent in atrial preparations from 5-HT_4_-TG than from H_2_-TG [32]. Hence, presumably due to the low intrinsic affinity of ergotamine in the ventricle of 5-HT_4_-TG but also in H_2_-TG and/or less effective coupling to signal transduction, no increase in the phosphorylation state of phospholamban could be noted. Fittingly, ergotamine (Table 5) failed to shorten the time of relaxation in the ventricle of H_2_-TG in contrast to ergometrine or LSD. Of note, under the same conditions, LSD increased the phosphorylation state of phospholamban in whole-heart homogenates from H_2_-TG and 5-HT_4_-TG but not in the homogenates from WT (Figure 4). In a previous study, it had been shown that psilocin increased the phosphorylation state of phospholamban in the atrium from 5-HT_4_-TG and in the human atrium [31]. Here, these studies are extended to the ventricle of 5-HT_4_-TG.

The effects of psilocin and psilocybin in the whole transgenic hearts were very likely 5-HT_4_ receptor-mediated because they are present only in 5-HT_4_-TG but not in their WT littermate. In addition, the same held true in the atrium: psilocin increased the force only in the atrium of 5-HT_4_-TG but not WT [31]. Moreover, psilocin and psilocybin augmented the phosphorylation state of phospholamban in the ventricle and atrium of 5-HT_4_-TG but not of wild-type hearts. Increased phosphorylation of phospholamban [36] can explain, at least in part, why psilocin and psilocybin reduced the time to relaxation and increased the rate of tension relaxation in ventricular preparations from 5-HT_4_-TG mice. Phosphorylated phospholamban has been demonstrated to increase the rate at which calcium cations are pumped from the cytosol into the sarcoplasmic reticulum in unit time. Thus, fewer calcium cations are present in the cytosol and can bind to the myofilaments, and myofilaments relax faster [35,36]. Furthermore, the present data that psilocybin, the prodrug of psilocin, is active in the ventricle of 5-HT_4_-TG is consistent with previous findings that psilocybin is active in the atrium of 5-HT_4_-TG [31]. The phosphorylation of the amino acid serine 16 is due to the activity of PKA [35]. Therefore, it can also be speculated that the PKA was activated by psilocin via 5-HT_4_ receptors and the cAMP formation. Such PKA-dependent phosphorylations have the potential to alter the calcium ion homeostasis within cardiomyocytes. Indeed, all cAMP-increasing agents are thought to increase the propensity of ventricular arrhythmias via such a mechanism. Hence, a translational prediction from this study could be that psilocin might lead to potentially lethal arrhythmias, and, thus, care should be taken when giving patients psilocin for psychiatric indications.

The action of psilocin can be compared with that of serotonin: 5-HT increased the phosphorylation state of phospholamban in the atrium of 5-HT_4_-TG and in isolated, perfused hearts of 5-HT_4_-TG, but not those of WT, and this led to arrhythmias [10]. Moreover, it was also shown that histamine via H_2_ histamine receptors increases the phosphorylation state of phospholamban in perfused hearts from H_2_-TG but not from WT [12]. Hence, this specific biochemical pathway from the H_2_ histamine receptor to the phosphorylation of phospholamban exists in the ventricle of H_2_-TG.

### 3.1. Clinical Relevance

In the human atrium, but also, of more relevance for the present study, in the human ventricle, H_2_ histamine receptors are present and mediate a PIE. This was reported in the very first paper on human hearts and H_2_ histamine receptors [37]. One could throw in that they only studied terminally failing hearts, and this might not hold true for non-failing hearts [37]. However, some years later, Du et al. (1994) had access to human ventricular preparations of non-failing hearts [38]. They reported a similar efficacy of histamine to increase the FOC via H_2_ receptors in the non-failing human ventricle and non-failing atrium [38], as in the studies in failing human ventricles [37,39,40]. Interestingly, in the porcine ventricle, the PIE to histamine is H_1_ histamine and not H_2_ histamine receptor-mediated [41], indicating that H_2_-TG is a useful predictor for the human ventricular function via human H_2_ histamine receptors and therefore was utilized in the present study. Hence, H_2_ histamine receptors are functional in normal human ventricles [1].

With the human ventricular 5-HT_4_ receptor, the situation is somewhat more subtle. It turned out that in the failing ventricle, 5-HT_4_ receptors are upregulated, perhaps as a compensatory mechanism to the downregulated β-adrenoceptors [3]. In such failing human ventricular preparations, serotonin exerted a PIE [3]. However, in ventricular preparations from non-failing hearts, serotonin failed to increase the FOC. This apparent discrepancy could be partially explained by the observation that serotonin can increase the FOC in the presence of phosphodiesterase inhibitors, even in non-failing human ventricular preparations [3,42]. The finding that a PIE was noted with LSD, psilocin, psilocybin, ergotamine, and ergometrine is of clinical relevance, given that the receptors on which these substances act are present in the human ventricle. However, whether this really occurs in patients needs to be elucidated in further studies. It could be achieved, for instance, by an infusion of agonists with or without antagonists in volunteers. However, the data presented here provides proof of principle for such an approach. Furthermore, the data support the observations in patients who exhibited an elevation in heart rate and blood pressure after taking psilocybin.

Another important aspect is the heteroregulation between histamine and serotonin, which influences, e.g., neuronal and cardiac activity. Histamine and serotonin exert complex and opposing effects on cardiac function, both directly through their own receptors and indirectly through heteroregulation. These interactions are important for normal cardiac function. Histamine can modulate serotonin release by acting on inhibitory H_3_ histamine receptors located on serotonin-producing neurons [43]. Serotonin has also been demonstrated to influence histamine release, though the specific mechanisms are less well-defined compared to histamine’s influence on serotonin. The interplay between histamine and serotonin via heteroreceptors (H_3_ for histamine on serotoninergic neurons, and potentially other receptors for serotonin on histaminergic neurons) is an important area of research [44].

### 3.2. Limitations

There are some limitations of the study that have to be mentioned: Firstly, the transferability of the mouse data to humans is at least partially questionable. The composition and density of the receptors in mouse and human cardiomyocytes are not comparable. Each transgenic mouse model is designed to represent the effects of a specific type of receptor, which is also much more strongly expressed in the mouse cardiomyocytes than in the human cardiomyocytes, in which all receptors are present together but at a lower density. Secondly, the concentration (dose) of psilocybin and psilocin tested here (10 µM) significantly exceeds the concentrations used in humans. After the administration of 25 mg psilocin, a dose commonly used in humans, the mean peak plasma concentration of the non-metabolized (psychoactive) psilocin was found to be approximately 0.1 µM [24]. Consequently, it can be assumed that cardiovascular side effects rarely occur at therapeutic doses of psilocybin or psilocin. The same applies to the psilocin-dependent prolongation of the QTc interval, which only becomes relevant at a psilocin concentration that is approximately three times higher than the expected psilocin concentration after administration of an oral psilocybin dose of 25 mg [23]. Thirdly, the crosstalk between neuronal and cardiac effects of the hallucinogens cannot be addressed in an experimental model based on isolated organs. Regrettably, no permits for the use of psychedelics in live animals were available, and such permits are currently very difficult to obtain from the local authorities due to animal welfare legislation. Finally, the interaction between histamine and serotonin via H_2_ and 5-HT_4_ receptors could not be investigated in this study. To address this point, double transgenic mice could be helpful, but with the continuing risk of questionable comparability with the human heart. However, this was beyond the scope of the study.

## 4. Materials and Methods

### 4.1. Transgenic Mice

Transgenic mice with cardiomyocyte-specific expression of the human 5-HT_4_ receptor (5-HT_4_-TG) and transgenic mice with cardiomyocyte-specific expression of the human H_2_ receptor (H_2_-TG) were generated and characterized previously [10,12]. The cardiac myocyte-specific expression was achieved by the use of the α-myosin heavy chain promoter. The breeding, housing, care, and supply of the mice were ensured by the faculty’s own animal facility. The mean age of the animals studied in the atrial contraction experiments was 223 days, and the group consisted of 40 male and 38 female mice. During the initial characterization of our transgenic mouse models, we also had a look for any sex differences in the response to receptor stimulation. However, we did not observe any differences between isolated cardiac preparations from male or female mice, at least in our transgenic models [10,12]. Therefore, we generally decided to perform all experiments on equal numbers of male and female mice. All mice were housed under conditions of optimum light, temperature, and humidity with food and water provided ad libitum. The animals were handled and maintained following the protocols given by the Animal Welfare Committee of the University of Halle-Wittenberg, Halle, Germany.

### 4.2. Western Blotting

The frozen heart was powdered at the temperature of liquid nitrogen in the presence of a sample buffer that inhibited proteolysis, phosphorylation, and dephosphorylation [10,12] using a mixer mill MM 400 (Retsch, Haan, Germany). The protein concentration of the homogenates was measured according to the Lowry method. Subsequently, aliquots of the homogenates containing 20 µg protein were run on sodium-dodecyl-sulfate gel electrophoresis. For the gel electrophoresis, commercially available precast gradient gels (Novex™ 4–20% Tris–Glycine Plus Midi Protein Gels; Thermo Fisher Scientific, Waltham, MA, USA) were utilized, and subsequently, the proteins were electrically transferred to nitrocellulose membranes via wet transfer (Amersham Protran 0.45 µm, Cytiva, München, Germany). Molecular weight markers were run and blotted as controls and then employed to cut membrane strips at the predicted molecular weight bands, as previously outlined. These small strips were incubated with primary antibodies directed against the phosphorylated form (serine 16) of phospholamban (Badrilla, Leeds, UK) or as a loading control against the cardiac form of calsequestrin (Abcam, Cambridge, UK). Thereafter, the membrane strips were incubated with horse radish-conjugated secondary antibodies, followed by the addition of a chemiluminescent substrate (Merck, Darmstadt, Germany). The chemiluminescent signals were detected with an Amersham ImageQuant800 imager (Cytiva Europe, Freiburg im Breisgau, Germany), and quantification was performed as described [10,12].

### 4.3. Langendorff Hearts

Isolated, spontaneously beating, retrogradely perfused mouse hearts were prepared according to the Langendorff method as described [12,32,34,45,46]. The heart was removed from the thoracic cavity and meticulously positioned on a cannula via the aorta. Custom-made equipment was built by the university workshop and utilized in this procedure. The hearts were retrogradely perfused using a peristaltic pump with a constant flow of 2 mL/min (controlled by a flow meter) with the buffer and allowed to beat spontaneously. Force was measured via a hook attached to the apex cordis. The hook was connected via a pulley to a force transducer. The composition of the modified Tyrode’s solution used here as buffer, the measurement of force from the left ventricular apex, the digitization of the electronical recordings (PowerLab system with LabChart 8 software; ADInstruments, Spechbach, Germany), and the freeze clamping of the hearts have been published by us [12]. In brief, the modified Tyrode’s solution contained (in mM) 119.8 NaCl, 5.4 KCI, 1.8 CaCl_2_, 1.05 MgCl_2_, 0.42 NaH_2_PO_4_, 22.6 NaHCO_3_, 0.05 Na_2_EDTA, 0.28 ascorbic acid, and 5.05 glucose. Here, the ascorbic acid was included as an antioxidant. Tyrode’s solution was continuously gassed with 95% O_2_ and 5% CO_2_ and maintained at 37 °C and pH 7.4 [12]. At the end of the perfusion, hearts were snap-frozen with Wollenberger clamps pre-cooled in liquid nitrogen. This frozen tissue was stored at −80 °C and then homogenized and used for the Western blots [10,12]. In this study, for all substances under consideration, a concentration was selected that has been previously determined to be the most effective concentration in isolated atria (ergometrine, 10 µM [34]; ergotamine, 10 µM [32]; LSD, 10 µM [33]; psilocin and psilocybin, 10 µM [31]).

### 4.4. Data Analysis

The data presented herein are expressed as means ± standard error of the mean. Student’s *t*-test was used for paired or unpaired samples as appropriate, and one-way ANOVA with Bonferroni correction was used for comparing more than two groups. This is stated in the appropriate legends to Figures or Tables. A probability value smaller than 0.05 was defined as significant in the present study.

### 4.5. Materials

The substances isoprenaline-bitartrate salt, serotonin (5-HT) hydrochloride, psilocin, ergometrine, and ergotamine were purchased from Sigma-Aldrich (Taufkirchen, Germany). Psilocybin and LSD were purchased from LGC (Luckenwalde, Germany). All other chemicals were of the highest purity grade commercially available. Deionized water was used throughout the experiments. Stock solutions were prepared fresh daily.

## 5. Conclusions

In summary, using the 5-HT_4_-TG model, it was possible to detect left ventricular inotropic effects of substances that are not intended to act on the heart, namely psilocin and psilocybin. Table 6 provides a schematic summary of the cardiac effects on the mouse heart and the human atrium of the compounds investigated either in this study or as referenced in the literature. Future implications: Based on the data presented here, it is clear that psilocybin and psilocin are, in principle, capable of inducing cardiovascular side effects in humans via 5-HT_4_ receptors. However, this is likely only relevant in cases of abuse or accidental overdose of psilocybin-containing preparations or in patients with an existing cardiovascular disease. Further studies in patients are needed to determine the optimal dosage of psilocybin that produces the desired antidepressant effect without (cardiovascular) side effects. Furthermore, interactions with other drugs that influence the serotoninergic system (e.g., antidepressants, which can also increase serotonin levels in the periphery) must be taken into account, especially as the combination of psilocybin with other centrally acting drugs is likely to occur more frequently in the future, whether intentionally or unintentionally.

## Figures and Tables

**Table 1:** Effect of psilocin on force of contraction (FOC), the time to peak tension (t1), the time of relaxation (t2), the maximum rate of contraction (dF/dt max) or the minimum rate of relaxation (dF/dt min), and the beating rate in Langendorff-perfused whole hearts.

Psilocin						
	WT		H_2_-TG		5-HT_4_-TG	
	Basal	After Psilocin	Basal	After Psilocin	Basal	After Psilocin
N	5		5		5	
Force (mN)	16.1 ± 2.3	17.8 ± 2.7	10.6 ± 0.7	12.0 ± 0.8 *	12.1 ± 2.2	18.4 ± 1.7 *
Rate of relaxation (mN/s)	−254.0 ± 28.2	−231.4 ± 28.1	−219.6 ± 12.8	−229.6 ± 11.3	−230.1 ± 35.8	−381.5 ± 38.7 *
Rate of tension development (mN/s)	395.7 ± 40.0	423.8 ± 40.6	315.2 ± 22.1	325.5 ± 21.1	323.3 ± 46.0	536.8 ± 58.5 *
Beating rate (bpm)	257.3 ± 32.9	231.4 ± 28.1	384.7 ± 24.6	347.8 ± 25.0	332.9 ± 31.5	275.0 ± 39.8 *
Time to peak tension (ms)	40.1 ± 2.2	42.5 ± 2.2	33.7 ± 0.9	37.6 ± 1.4	35.1 ± 2.7	35.8 ± 3.4
Time of relaxation (ms)	65.3 ± 5.2	70.9 ± 5.5	48.7 ± 2.4	52.0 ± 2.8	51.9 ± 2.4	52.7 ± 6.9

* *p* < 0.05 vs. basal values (before the addition of substances) in Student’s paired *t*-test. N is the number of animals.

**Table 2:** Effect of psilocybin on force of contraction (FOC), the time to peak tension (t1), the time of relaxation (t2), the maximum rate of contraction (dF/dt max) or the minimum rate of relaxation (dF/dt min), and the beating rate in Langendorff-perfused whole hearts.

Psilocybin						
	WT		H_2_-TG		5-HT_4_-TG	
	Basal	After Psilocybin	Basal	After Psilocybin	Basal	After Psilocybin
N	5		5		5	
Force (mN)	15.5 ± 2.2	19.6 ± 3.1	13.2 ± 1.6	14.7 ± 1.7*	12.2 ± 1.2	16.7 ± 1.6 *
Rate of relaxation (mN/s)	−269.1 ± 48.8	−354.2 ± 65.0	−281.9 ± 35.0	−298.9 ± 39.9	−263.4 ± 38.1	−393.3 ± 52.2 *
Rate of tension development (mN/s)	399.4 ± 49.1	515.9 ± 78.3	431.3 ± 43.9	432.7 ± 49.8	375.8 ± 41.3	521.7 ± 53.4 *
Beating rate (bpm)	253.8 ± 17.7	246.1 ± 13.3	364.5 ± 37.0	333.2 ± 44.9	332.6 ± 36.9	312.9 ± 36.7
Time to peak tension (ms)	38.1 ± 1.9	38.5 ± 1.5	32.8 ± 1.7	36.8 ± 2.0	32.9 ± 0.4	32.7 ± 1.2
Time of relaxation (ms)	65.0 ± 6.4	60.3 ± 0.9	48.5 ± 4.2	51.5 ± 5.7	49.2 ± 4.3	45.8 ± 4.0

* *p* < 0.05 vs. basal values (before the addition of substances) in Student’s paired *t*-test. N is the number of animals.

**Table 3:** Effect of LSD on force of contraction (FOC), the time to peak tension (t1), the time of relaxation (t2), the maximum rate of contraction (dF/dt max) or the minimum rate of relaxation (dF/dt min), and the beating rate in Langendorff-perfused whole hearts. For comparison, the data from WT were retabulated from [33].

LSD						
	WT		H_2_-TG		5-HT_4_-TG	
	Basal	After LSD	Basal	After LSD	Basal	After LSD
N	5		6		5	
Force (mN)	12.6 ± 2.7	14.9 ± 3.1	8.8 ± 1.5	15.1 ± 2.0 *	13.6 ± 2.5	16.3 ± 2.7 *
Rate of relaxation (mN/s)	−176.2 ± 38.7	−186.3 ± 38.5	−259.5 ± 60.2	−363.5 ± 82.9 *	−243.3 ± 49.4	−325.1 ± 74.8
Rate of tension development (mN/s)	298.8 ± 64.0	330.6 ± 66.2	381.9 ± 26.2	252.0 ± 104.1 *	360.1 ± 60.9	475.9 ± 94.4 *
Beating rate (bpm)	219.6 ± 20.8	193.1 ± 9.5	318.6 ± 26.2	293.7 ± 17.8	297.7 ± 29.1	275.6 ± 22.7 *
Time to peak tension (ms)	41.9 ± 1.8	43.8 ± 1.9	32.1 ± 1.2	29.7 ± 0.5 *	36.3 ± 1.6	35.3 ± 2.3
Time of relaxation (ms)	78.1 ± 4.1	85.0 ± 1.6	61.7 ± 5.3	47.7 ± 3.3	59.4 ± 3.1	66.1 ± 11.3

* *p* < 0.05 vs. basal values (before the addition of substances) in Student’s paired *t*-test. N is the number of animals.

**Table 4:** Effect of ergometrine on force of contraction (FOC), the time to peak tension (t1), the time of relaxation (t2), the maximum rate of contraction (dF/dt max) or the minimum rate of relaxation (dF/dt min), and the beating rate in Langendorff-perfused whole hearts. For comparison, the data from WT and 5-HT_4_-TG were retabulated from [34].

Ergometrine						
	WT		H2-TG		5-HT4-TG	
	Basal	After Ergometrine	Basal	After Ergometrine	Basal	After Ergometrine
N	5		6		5	
Force (mN)	12.9 ± 1.4	13.2 ± 1.4	7.9 ± 1.6	14.6 ± 2.8 *	10.1 ± 2.9	11.6 ± 3.2
Rate of relaxation (mN/s)	−201.4 ± 22.4	−205.0 ± 21.2	−133.5 ± 20.8	−310.7 ± 45.0 *	−167.7 ± 45.5	−192.7 ± 55.0
Rate of tension development (mN/s)	253.5 ± 34.3	352.3 ± 34.3	219.6 ± 30.2	446.7 ± 52.7 *	271.7 ± 72.9	332.4 ± 96.7
Beating rate (bpm)	258.5 ± 19.6	271.1 ± 15.0	370.3 ± 18.8	362.3 ± 27.3	294.6 ± 31.7	291.2 ± 23.0
Time to peak tension (ms)	38.2 ± 2.0	38.9 ± 2.2	33.2 ± 2.4	30.2 ± 1.3	34.6 ± 2.5	35.0 ± 2.6
Time of relaxation (ms)	68.0 ± 5.4	67.5 ± 6.3	56.0 ± 3.6	45.8 ± 3.8 *	61.9 ± 4.8	65.3 ± 5.1

* *p* < 0.05 vs. basal values (before the addition of substances) in Student’s paired *t*-test. N is the number of animals.

**Table 5:** Effect of ergotamine on force of contraction (FOC), the time to peak tension (t1), the time of relaxation (t2), the maximum rate of contraction (dF/dt max) or the minimum rate of relaxation (dF/dt min), and the beating rate in Langendorff-perfused whole hearts. For comparison, the data from WT were retabulated from [32].

Ergotamine						
	WT		H_2_-TG		5-HT_4_-TG	
	Basal	After Ergotamine	Basal	After Ergotamine	Basal	After Ergotamine
N	5		7		5	
Force (mN)	12.8 ± 1.1	13.8 ± 1.2	10.6 ± 1.5	14.7 ± 1.9 *	12.5 ± 3.2	15.5 ± 4.2
Rate of relaxation (mN/s)	−243.9 ± 9.0	−268.5 ± 18.0	−196.7 ± 26.6	−292.5 ± 43.9 *	−183.4 ± 40.6	−233.1 ± 56.0
Rate of tension development (mN/s)	337.6 ± 34.4	368.1 ± 39.4	329.2 ± 46.4	435.0 ± 54.9 *	298.2 ± 62.3	395.6 ± 93.4
Beating rate (bpm)	262.2 ± 11.3	254.0 ± 8.7	352.7 ± 23.2	296.5 ± 41.3	279.5 ± 17.0	279.2 ± 17.9
Time to peak tension (ms)	44.5 ± 7.3	45.2 ± 7.3	31.8 ± 1.3	34.6 ± 2.0	35.5 ± 2.8	38.0 ± 2.7
Time of relaxation (ms)	57.2 ± 4.3	54.9 ± 6.2	53.7 ± 2.5	62.6 ± 11.9	70.8 ± 5.6	68.5 ± 4.5

* *p* < 0.05 vs. basal values (before the addition of substances) in Student‘s paired *t*-test. N is the number of animals.

**Table 6:** Synopsis of the effect (↑ large increase, + small increase, − no effect) of psilocin, psilocybin, ergotamine, ergometrine, LSD, and for comparison, serotonin and histamine, on force of contraction (FOC) in human atrial preparation (HAP), human ventricular preparations (HVP), isolated atrium, or isolated perfused whole heart from WT, H_2_-TG, and 5-HT_4_-TG. nd: not done.

	HAP	HVP	WT Atrium	WT Whole Heart	H_2_-TG Atrium	H_2_-TG Whole Heart	5-HT_4_-TG Atrium	5-HT_4_-TG Whole Heart
LSD	↑ ^5^	nd	− ^5^	− ^3,5^	↑ ^5^	↑ ^3^	↑ ^5^	↑ ^3^
Ergotamine	↑ ^7^	nd	− ^7^	− ^3,7^	↑ ^7^	↑ ^3^	+ ^7^	− ^3^
Ergometrine	↑ ^6^	nd	− ^6^	− ^3,6^	↑ ^6^	↑ ^3^	− ^6^	− ^3^
Psilocin	↑ ^4^	nd	− ^4,10^	− ^3^	nd	− ^3^	↑ ^10^	↑ ^3^
Psilocybin	↑ ^4^	nd	− ^4,10^	− ^3^	nd	− ^3^	↑ ^10^	↑ ^3^
5-HT	↑ ^1^	↑ ^1^	− ^4,8^	− ^3,8^	nd	nd	↑ ^1,8^	↑ ^1,8^
Histamine	↑ ^2^	↑ ^2^	− ^4,9^	− ^3,9^	↑ ^2,9^	↑ ^2,9^	nd	nd

^1^ [44], ^2^ [47], ^3^ this paper, ^4^ [9],^5^ [33], ^6^ [34], ^7^ [32], ^8^ [10], ^9^ [12], ^10^ [31].

## Data Availability

The raw data supporting the conclusions of this article will be made available by the authors on request. The data are not publicly available due to legal reasons.

## Abbreviations

The following abbreviations are used in this manuscript:

FOC	Force of contraction
LSD	Lysergic acid diethylamide
PIE	Positive inotropic effect
PKA	Protein kinase A
WT	Wild type
